# Enhanced Design and Characterization of a Wearable IMU for High-Frequency Motion Capture

**DOI:** 10.3390/s25196224

**Published:** 2025-10-08

**Authors:** Diego Valdés-Tirado, Gonzalo García Carro, Juan C. Alvarez, Diego Álvarez, Antonio López

**Affiliations:** Multisensor Systems and Robotics Group (SiMuR), Department of Electrical, Electronic, Communications and Systems Engineering, University of Oviedo, 33204 Gijón, Spain

**Keywords:** wearable IMU, gait analysis, inertial sensors, energy efficiency, signal characterization, embedded systems

## Abstract

This paper presents the third-generation design of Bimu, a compact wearable inertial measurement unit (IMU) tailored for advanced human motion tracking. Building on prior iterations, Bimu R2 focuses on enhancing thermal stability, data integrity, and energy efficiency by integrating onboard memory, redesigning the power management system, and optimizing the communication interfaces. A detailed performance evaluation—including noise, bias, scale factor, power consumption, and drift—demonstrates the device’s reliability and readiness for deployment in real-world applications ranging from clinical gait analysis to high-speed motion capture. The improvements introduced offer valuable insights for researchers and engineers developing robust wearable sensing solutions.

## 1. Introduction

Wearable inertial measurement units (IMUs) have become indispensable tools for assessing human motion in health monitoring, rehabilitation, sports biomechanics, and other applied fields. Their low cost, portability, and capacity for long-term data acquisition outside laboratory settings have enabled new opportunities in the detection of early functional decline, fall risk prediction, movement pattern analysis, and performance optimization [1,2,3,4].

However, these same features introduce technical trade-offs that still limit the broader impact of IMU-based systems, particularly in scenarios requiring high precision, long-term autonomy, or resilience under dynamic conditions [5,6]. As several studies have highlighted, a critical barrier to the clinical and translational adoption of wearable IMUs is the lack of standardization and rigor in device characterization [7,8]. Variations in sampling fidelity, thermal drift, mechanical stability, and energy efficiency can significantly affect the reliability of recorded signals, especially in applications where small deviations—such as step-to-step gait variability, transient kinematic events, or high-impact athletic maneuvers—hold clinical significance [9,10]. Furthermore, the increasing demand for continuous monitoring in real-world environments calls for devices that combine laboratory-grade measurement performance with robustness, miniaturization, and low power consumption [4,11].

Energy efficiency and temperature sensitivity are particularly critical factors in modern wearable IMU design, as both directly impact autonomy and signal stability [5]. Moreover, high sampling rates (≥500 Hz) are often required to accurately capture impact dynamics in sports and rapid kinematic events, which has been shown to affect the accuracy of tibial acceleration and related parameters [12,13]. In demanding applications, wearable IMUs must meet stringent specifications to ensure reliability and accuracy. These requirements include (i) energy efficiency to maximize autonomy in long-term deployments by reducing unnecessary power losses [5]; (ii) high sampling frequency and stable performance to capture rapid kinematic events with fidelity [4]; (iii) thermal management to minimize temperature-induced bias and drift, thereby reducing measurement error [6]; and (iv) structural robustness, where improved PCB rigidity enhances signal stability and measurement precision [14]. Meeting these specifications directly translates into greater usability of IMUs in clinical, sports, and field scenarios. In this context, Bimu R2 has been specifically designed to address these needs, with architectural improvements that combine power efficiency, thermal stability, high-frequency data acquisition, and mechanical reinforcement for precision sensing. Several commercial platforms have contributed significantly to wearable motion analysis, including NilsPod [15], Shimmer 3 [16], Physilog^®^5 [17], and XSens Dot [18]. While each system provides valuable features—such as long-term recording (NilsPod), wireless connectivity (Shimmer 3), or clinical-grade validation (Physilog^®^5)—they also exhibit limitations in synchronization accuracy, thermal drift stability, or resilience in high-frequency scenarios. These constraints have motivated the design of Bimu R2, which explicitly targets energy efficiency, thermal stability, robust synchronization, and reliable high-frequency acquisition as critical differentiators.

In the field of athletic performance and impact biomechanics, the need for high-fidelity motion data has grown increasingly urgent. Traditional laboratory-based motion capture systems, while accurate, are constrained by their immobility, high cost, and susceptibility to occlusion. In contrast, wearable IMUs offer a portable and cost-effective alternative, enabling continuous monitoring in real-world environments. These advantages are particularly beneficial in sports contexts, where capturing high-resolution kinematic data during dynamic activities—such as sprinting, jumping, or contact events—is essential for performance optimization and injury prevention.

Current motion analysis approaches used in elite training environments often fail to capture biomechanical parameters in real competition conditions, limiting their ecological validity. The capacity to monitor athletes on actual tracks, during realistic training sessions or competitive events, is critical for generating actionable insights into stride dynamics, acceleration phases, and ground contact mechanics—key elements in performance evaluation and technical refinement. However, existing commercial IMUs often fall short in scenarios requiring high-frequency data acquisition and precise synchronization—limitations that are especially critical in impact studies and sprinting biomechanics, where rapid force transfers and neuromuscular responses must be captured in millisecond intervals.

These combined challenges are the foundation for the initial development of Bimu, a compact wearable platform designed to bridge the gap between high-frequency motion capture and field-deployable inertial sensing. Building upon previous versions presented in academic and technical forums [14,19], this work represents the latest evolution of the Bimu system, responding to critical limitations observed in early deployments and aligning with the needs of both demanding biomechanical research and high-performance athletic monitoring.

## 2. Hardware Design Enhancements for Bimu R2

**Figure 1:**
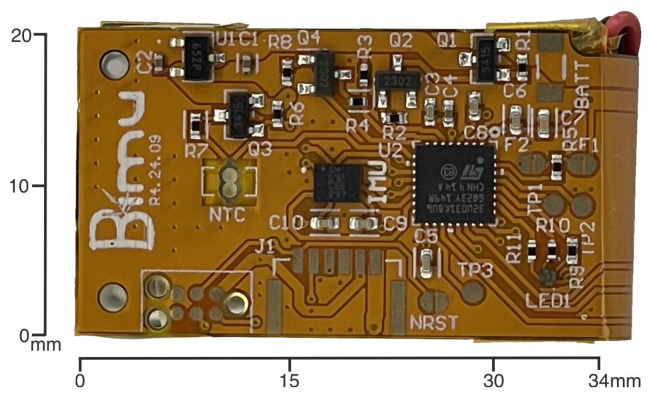
Top view of the Bimu R2 PCB showing the STM32U031K4 microcontroller and supporting circuitry. The compact layout integrates the inertial sensor, power regulation, and communication interfaces.

**Figure 2:**
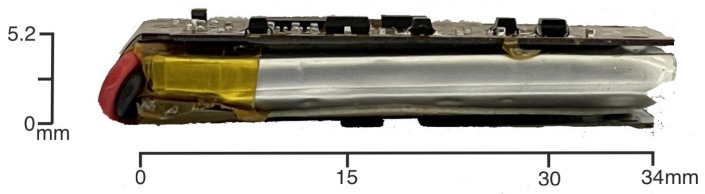
Side view of the Bimu R2 prototype illustrating the integration of the rechargeable Li-Po battery within the stacked assembly.

**Figure 3:**
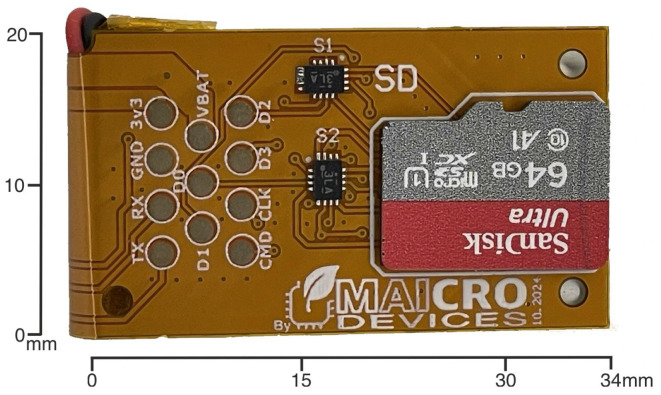
Bottom view of the PCB highlighting the microSD socket and additional control circuitry. This revision integrates memory and power management features into a compact footprint.

**Figure 4:**
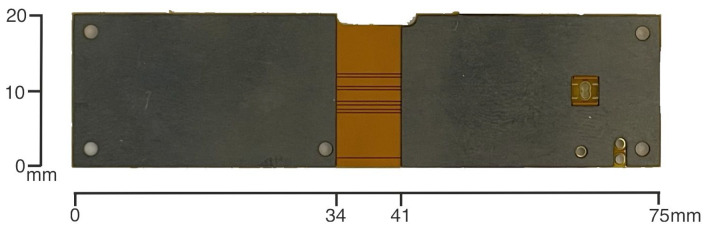
Rear view of the assembled device, including the 0.3 mm stainless-steel stiffener used to increase mechanical stability and thermal robustness.

To enhance the overall performance, mechanical robustness, and energy efficiency of the inertial measurement unit (IMU) platform, several critical improvements are introduced in the latest design iteration. These include (1) an upgrade of the microcontroller unit (MCU), (2) mechanical reinforcement via the addition of a 0.3 mm steel stiffener to the flexible PCB, (3) a simplification of the power management strategy by externalizing the battery management system (BMS), and (4) the removal of the external microSD card interface in favor of an integrated memory system with a unified I/O and charging socket. Each of these modifications is detailed below, see Figure 1, Figure 2, Figure 3 and Figure 4.

### 2.1. Microcontroller Replacement: From STM32L051 to STM32U031

A key improvement in the updated design is the replacement of the STM32L051 (STMicroelectronics N.V., Geneva, Switzerland) microcontroller with the more modern STM32U031 (STMicroelectronics N.V., Geneva, Switzerland). The STM32U0 (STMicroelectronics N.V., Geneva, Switzerland) series introduces significant benefits in terms of power efficiency, processing capability, and peripheral integration, which are especially advantageous in IMU-based systems requiring real-time data acquisition and low-power operation. A comparative summary of relevant features is provided in Table 1.

The STM32U031’s enhanced RAM and lower power consumption allow for more efficient buffering of IMU data and extended operation in energy-constrained environments. Furthermore, the upgraded low-power modes significantly reduce idle energy usage, which is critical for long-term deployments.

### 2.2. Steel Stiffener Integration for Mechanical and Thermal Stability

**Figure 5:**
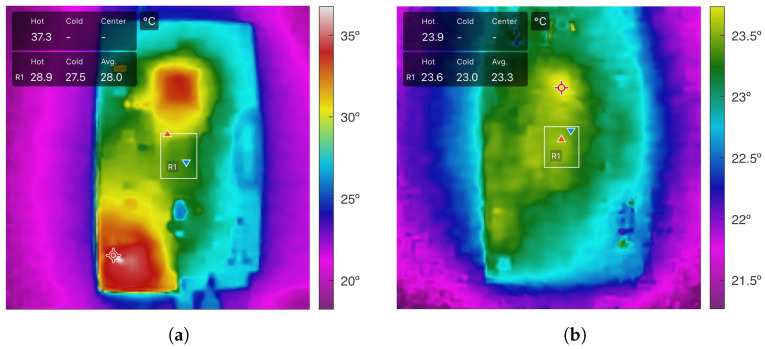
Thermal comparison between Bimu r1.5 (FR4) and Bimu R2 (steel stiffener). The color bar on the right indicates the temperature scale in °C, with warmer colors (red/yellow) representing higher temperatures and cooler colors (blue/green) indicating lower temperatures. (**a**) Bimu r1.5 with 0.8 mm FR4 substrate. (**b**) Bimu R2 with 0.3 mm steel stiffener.

To address mechanical vulnerabilities inherent to flexible PCB-based designs, a 0.3 mm stainless steel stiffener is introduced in the region where the IMU is mounted. This structural enhancement offers the following benefits:

- Vibration and Mechanical Noise Suppression: The stiffener reduces flex-induced noise and mechanical resonance, improving the fidelity of accelerometer and gyroscope measurements.
- Thermal Dissipation: Acting as a passive heat spreader, the stiffener helps maintain thermal stability, reduces temperature-induced drift, and extends component life. Compared to an FR4 substrate of 0.8 mm, which offers higher rigidity but lower thermal conductivity, the 0.3 mm steel stiffener enables more favorable heat spreading. Experimental thermal imaging in our conference paper [19] showed an approximate 5 °C reduction at the IMU under continuous operation (Figure 5).
- Impact Resistance: It provides structural reinforcement, protecting the IMU from shocks, deformation, and handling stresses during assembly or field use.
- Signal Integrity Improvement: Better mechanical alignment of the flexible PCB enhances contact reliability, reduces EMI, and ensures more accurate and stable sensor data.

Despite a slight increase in weight and manufacturing complexity, the steel stiffener proved to be a highly effective solution for improving mechanical, thermal, and signal integrity without compromising compactness. Although mechanical rigidity tests were not conducted in the present study, the improved thermal behavior justified the adoption of the stiffener. Future work will further investigate the balance between stiffness and thermal performance.

### 2.3. Power Architecture Simplification

In the updated design, the power management system is restructured by externalizing the Battery Management System (BMS) and employing a dedicated LDO regulator for voltage regulation on-board. This approach allows for the following:

- **Reduced PCB Complexity:** Removing the integrated BMS frees up board space and simplifies the layout.
- **Improved Regulation Efficiency:** A low-noise LDO ensures stable and clean power delivery to sensitive analog and digital components.
- **Modular Integration:** The external BMS can be customized separately, enabling system-level design flexibility.

This simplification aligns with low-power design principles and improves overall modularity while maintaining electrical and thermal performance.

### 2.4. Integrated Memory and Multifunction Connector

Another significant enhancement is the integration of onboard memory. In the updated design, the external microSD socket previously used for data logging has been removed, and a microSD memory device is now directly soldered onto the PCB. This change improves device compactness, mechanical robustness, and enclosure hermeticity, while still providing high-capacity storage for long-term data acquisition.

To complement this change, a custom-designed multifunction I/O socket is implemented. This socket serves both as a data communication interface and as a charging port for the onboard battery, simplifying the external connections required for operation. The unified connector supports firmware flashing, memory readout, and battery charging through a single port, reducing connector footprint and improving mechanical durability. This change enhances system integration, reduces connector wear, and simplifies deployment in embedded applications.

## 3. Methodology

To evaluate the performance of the new version of the Bimu, a series of controlled experiments are conducted. The methodology consisted of dynamic rotation tests to assess sensor response, static tests for noise characterization, and power consumption measurements. Each aspect of the experimental setup is detailed below.

### 3.1. Power Consumption Measurement

Power consumption is measured to assess the IMU’s energy usage in various operating modes. The custom IMU board’s supply line is instrumented with an INA228 precision current sensor (Texas Instruments, Dallas, USA) interfaced to an ESP32 microcontroller (Espressif Systems, Shanghai, China) for data acquisition. The INA228 is a 20-bit shunt-based power monitor, enabling high-resolution measurement of current draw. During testing, the ESP32 sampled the IMU’s current consumption at 1 kHz (1000 samples per second) and streamed these measurements to a PC for logging and analysis. This high sampling rate ensured that even rapid transients in current (for example, spikes during sensor readout or processing bursts) are captured. Measurements are conducted for each IMU operating mode under consideration, including different sensor ODRs (output data rates) and power configurations (such as high-performance vs. low-power settings of the LSM6DSL (STMicroelectronics, Geneva, Switzerland). For each mode, the IMU is allowed to run through its normal data acquisition cycle while the current is recorded. The analysis of the logged power data focused on identifying the current consumption patterns across different acquisition phases of the device’s operation. In particular, the current profile is examined for distinct phases such as idle periods, active sampling/measurement intervals, and any data processing or transmission events. By segmenting the power trace into these phases, the contribution of each operation stage to overall energy usage is determined. This methodology allowed the characterization of both the average power consumption in each mode and the peak currents associated with transient events, providing a comprehensive understanding of the IMU’s energy requirements for various use cases. The results from these power measurements are crucial for evaluating battery life and ensuring the system meets low-power design goals.

### 3.2. Static Test Setup

**Figure 6:**
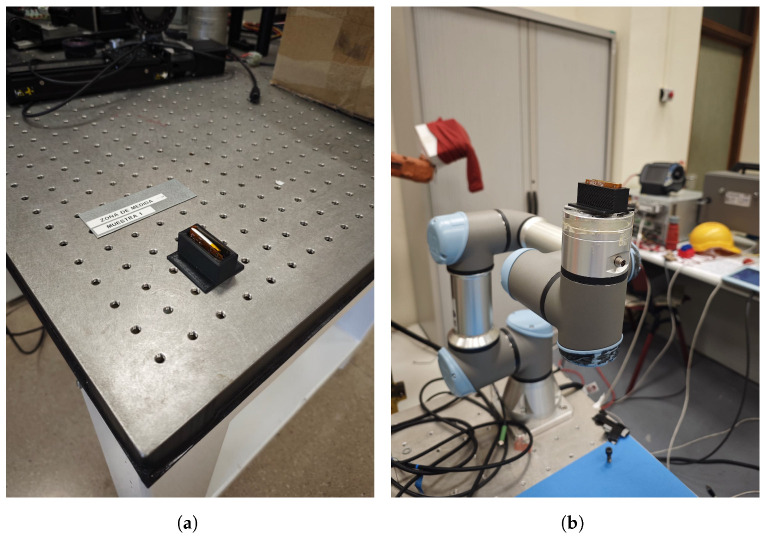
Experimental setups. (**a**) Sensor placed on the vibration-isolated table for static measurement experiment. (**b**) Sensor placed on the UR3 robotic arm for dynamic measurement experiment.

For the stationary noise characterization, the IMU is evaluated in a completely static condition using a specialized mounting setup. A custom 3D-printed support is designed to hold the IMU rigidly in a fixed orientation, ensuring precise alignment of the sensor’s axes (e.g., one accelerometer axis aligned with the gravity vector) during the test. This mount is placed on an anti-vibration table (Figure 6a) to isolate the sensor from external micro-vibrations and environmental disturbances (such as floor vibrations or air currents). In this static configuration, the IMU remained at rest while its output is sampled continuously over an extended duration. The collected stationary data enabled calculation of the inherent sensor bias (offset) and noise level. In particular, the mean bias and standard deviation (STD) of the accelerometer and gyroscope signals are computed to quantify static accuracy and short-term noise, respectively. Additionally, an Allan deviation analysis is performed on the long stationary data sequences to characterize the IMU’s stochastic noise behavior over varying integration times. The Allan deviation yields insight into noise processes such as bias instability and random walk by examining how the variance of the sensor output evolves over different averaging intervals. These static tests, conducted under carefully controlled conditions, provided a baseline measurement of the sensor’s inherent drift and noise floor, which is critical for understanding its performance in the absence of motion.

### 3.3. Dynamic Test Setup

For dynamic response characterization, the IMU module is mounted on a UR3 robotic arm (Universal Robots, Denmark) [19] (Figure 6b), which provided precise and repeatable motion (up to ±0.1 mm positional repeatability). The robotic arm is programmed to rotate the sensor about each of its principal axes (X, Y, and Z) in separate trials, allowing one-axis-at-a-time excitation. Each rotation is executed at a constant angular velocity of 75°/s, chosen to represent a moderate dynamic motion within the IMU’s measurement range. During each trial, the rotation is maintained for 15 s, and the IMU’s raw accelerometer and gyroscope outputs are recorded continuously via the onboard STM32U031 microcontroller. This procedure is repeated for each principal axis under a variety of IMU configurations (covering the range of sampling frequencies and full-scale sensitivity settings supported by the LSM6DSL). By testing multiple data rates and sensitivity scales, the dynamic behavior of the sensor (e.g., scale factor accuracy) could be evaluated under different operating conditions. The use of the UR3 arm ensured a constant rotation rate and consistent rotation angle, providing a ground-truth motion profile for subsequent error analysis. It is important to note that all experimental results reported in this work were obtained using the upgraded STM32U031. Results from the previous version of the device, based on the STM32L051, have already been published in our earlier study [14] and are referenced here solely for comparative purposes.

## 4. Results

### 4.1. Power Consume

**Figure 7:**
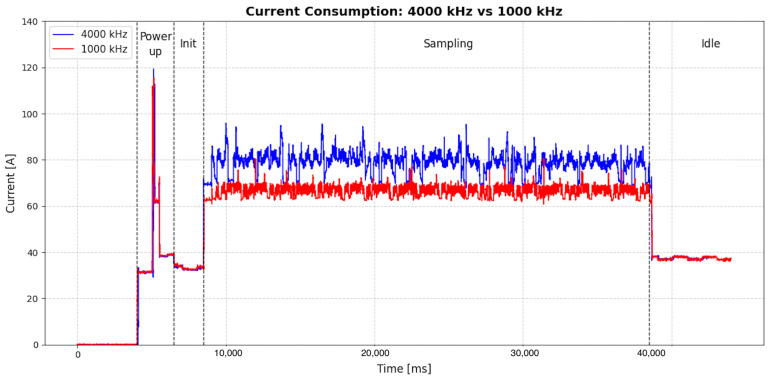
Current consume.

From the power profiling results, as shown in Figure 7, practical battery lifetime can be estimated. Using the integrated 220 mAh Li-Po cell, continuous operation at a 4000 Hz sampling rate reduces runtime to about 2 h and 49 min. For comparison, commercial alternatives such as the Shimmer3 (250 mAh, 6 h at 1024 Hz) and the Xsens Dot (180 mAh, 4 h at 1200 Hz) offer shorter durations under less demanding conditions.

### 4.2. Standard Deviation

**Figure 8:**
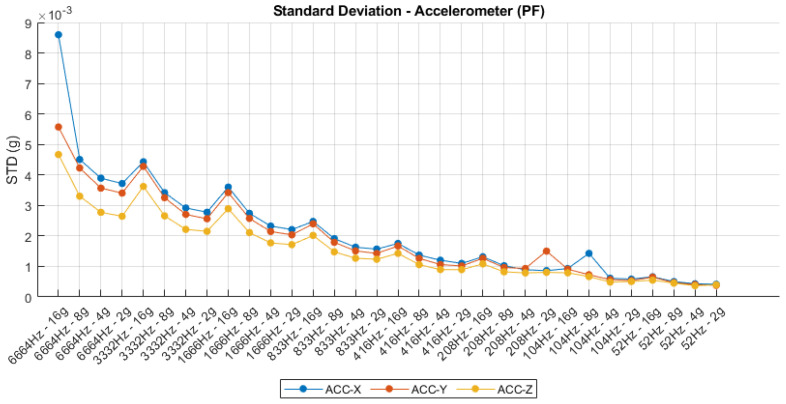
Accelerometer standard deviation.

**Figure 9:**
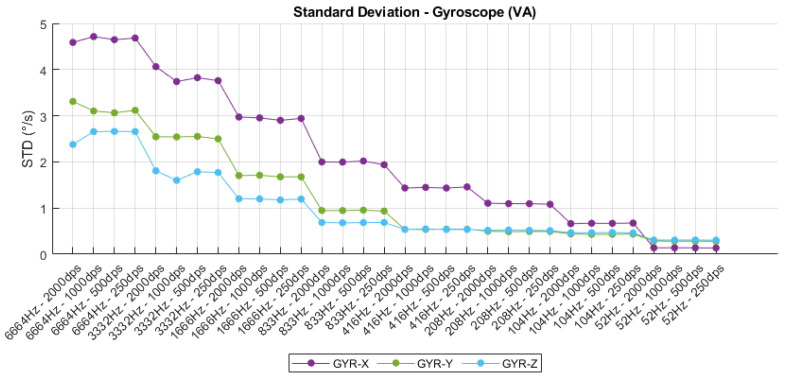
Gyroscope standard deviation.

The standard deviation analysis conducted across varying sampling frequencies and sensitivity configurations reveals critical insights into the noise characteristics of the IMU. For the accelerometer, as shown in Figure 8, a clear correlation is observed between sampling frequency and output variability, with higher frequencies (e.g., 6664 Hz) yielding greater standard deviations across all axes, particularly on the X-axis. This behavior is consistent with the increased bandwidth and susceptibility to high-frequency noise at elevated sampling rates. Furthermore, lower sensitivity settings (e.g., ±2 g) consistently demonstrate reduced standard deviation values, indicating higher precision in low-dynamic scenarios. In contrast, the gyroscope data depicted in Figure 9 exhibit a similar trend: standard deviation decreases progressively with reduced sampling frequency and higher sensitivity ranges. The GYR-X axis, in particular, shows significantly higher noise levels compared to the Y and Z axes across all configurations, suggesting potential axis-specific imperfections or calibration imbalances. These findings underscore the importance of careful selection of sampling parameters in IMU-based instrumentation systems, as they directly influence signal quality and measurement fidelity.

### 4.3. Drift

To evaluate the long-term stability of the inertial measurement unit (IMU), a drift analysis is conducted using a continuous dataset spanning 2500 s at a sampling rate of 3332 Hz. The drift analysis was performed using a single long-term dataset per version. The drift is estimated by fitting a first-order polynomial (linear regression) to each inertial axis of both the accelerometer and gyroscope signals. The slope of this fit, expressed in units of g/s^2^ for acceleration and °/s^2^ for angular velocity, is interpreted as the drift rate. The drift values reported here were estimated from the raw, unfiltered accelerometer and gyroscope signals, ensuring that the results directly reflect the intrinsic long-term stability of the sensor.

**Figure 10:**
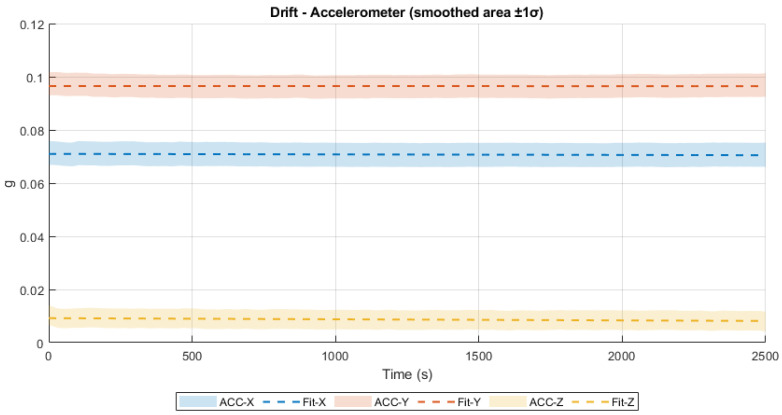
Accelerometer drift.

The resulting drift values for the accelerometer are −2.03 × 10^−7^ g/s^2^ for the X axis, −2.29 × 10^−8^ g/s^2^ for the Y axis, and −4.21 × 10^−7^ g/s^2^ for the Z axis. These values reflect highly stable behavior over time and are considered negligible with respect to the IMU’s resolution and dynamic range. The corresponding drift trends are illustrated in Figure 10, where the measured signals and their linear approximations are shown for all three axes. The flatness of the curves supports the numerical results and indicates excellent accelerometer performance in static conditions.

**Figure 11:**
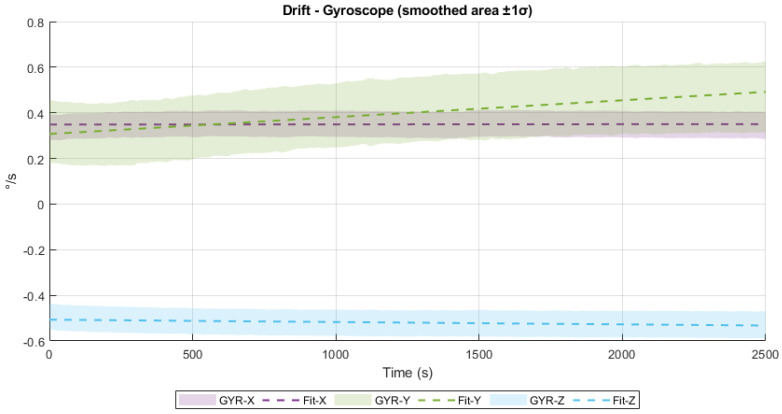
Gyroscope drift.

In the case of the gyroscope, the drift rates are $7.94\times 10^{-7}$°/s^2^ (X axis), $7.39\times 10^{-5}$°/s^2^ (Y axis), and $-1.03\times 10^{-5}$°/s^2^ (Z axis). While the X and Z axes exhibit minimal drift, the Y axis shows a slightly more pronounced linear trend over time. Nevertheless, even the highest observed drift (Y axis) would accumulate only about 0.185°/s over the 2500 s interval, which remains within acceptable limits for typical applications not requiring high-precision inertial navigation. The drift behavior of the gyroscope is presented in Figure 11, clearly showing the linear tendencies and confirming the stability of the system.

A direct comparison between the current drift measurements (corresponding to the R2 version of the sensor) and those obtained from the previous R1 version reveals a noticeable improvement in performance, particularly for the accelerometer. In R1, the drift values are $-4.52\times 10^{-7}$ g/s^2^ (X), $2.77\times 10^{-6}$ g/s^2^ (Y), and $2.51\times 10^{-7}$ g/s^2^ (Z). In contrast, the R2 sensor exhibited reduced drift on all axes: $-2.03\times 10^{-7}$ g/s^2^ (X), $-2.29\times 10^{-8}$ g/s^2^ (Y), and $-4.21\times 10^{-7}$ g/s^2^ (Z). Most notably, the Y-axis drift decreased by nearly two orders of magnitude, suggesting improved thermal stability or reduced electronic noise in the newer design.

Regarding the gyroscope, the R2 version shows comparable drift levels to R1. While the X- and Z-axis drift values decreased slightly (from $4.50\times 10^{-5}$°/s^2^ to $7.94\times 10^{-7}$°/s^2^ on X, and from $2.19\times 10^{-5}$°/s^2^ to $-1.03\times 10^{-5}$°/s^2^ on Z), the Y-axis drift increased from $-4.51\times 10^{-5}$°/s^2^ to $7.39\times 10^{-5}$°/s^2^. This increase, although still within acceptable bounds, could be attributed to alignment differences or slight calibration mismatches during the test.

Overall, the R2 version of the IMU demonstrates a clear enhancement in accelerometer drift stability and maintains gyroscope drift within acceptable performance margins. These findings support the conclusion that R2 provides a more stable baseline for long-term inertial measurements.

The drift results are presented as descriptive values, with the most notable improvement observed on the Y-axis, where the R2 version exhibited a reduction of nearly two orders of magnitude compared to R1. Overall, the R2 IMU demonstrates a marked enhancement in accelerometer drift stability while maintaining gyroscope drift within acceptable performance margins. These findings indicate that R2 provides a more stable and reliable baseline for long-term inertial measurements.

### 4.4. Fixed Bias

**Figure 12:**
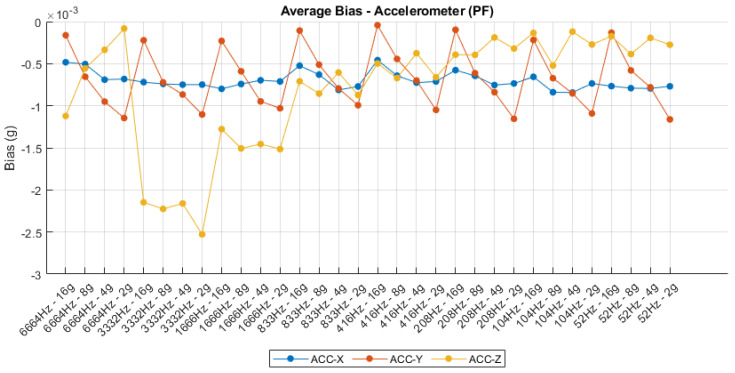
Accelerometer fixed bias.

**Figure 13:**
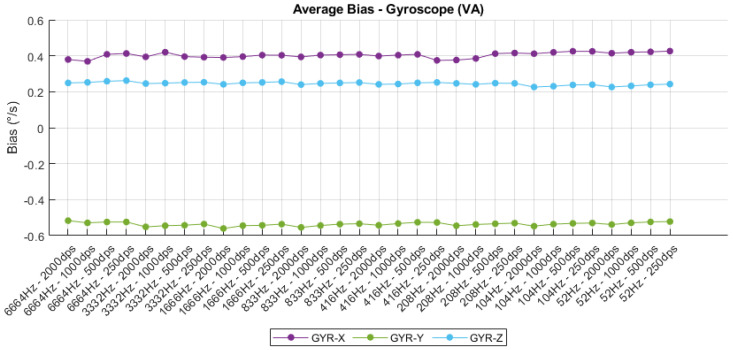
Gyroscope fixed bias.

The bias analysis of both accelerometer and gyroscope outputs, illustrated in Figure 12 and Figure 13, respectively, highlights the static offset behavior under varying sampling rates and sensitivity settings. For the accelerometer, the average bias remains relatively stable along the X and Y axes, with values oscillating around −0.8 mg and 1 mg, respectively. However, the Z-axis shows more significant fluctuations, particularly at lower sampling frequencies and sensitivity levels, reaching deviations close to −2.7 mg. These variations may be attributed to sensor orientation errors, mechanical asymmetries, or temperature sensitivity, and suggest the necessity of axis-specific calibration for applications requiring high absolute accuracy. Conversely, the gyroscope bias demonstrates exceptional consistency across all configurations, with the X- and Z-axes maintaining steady positive offsets around 0.4 dps and 0.3 dps, while the Y-axis exhibits a minor negative bias near −0.5 dps. The minimal variation across configurations confirms the gyroscope’s strong thermal and structural stability, although the presence of fixed bias across all axes indicates the need for post-processing compensation or factory-level trimming to minimize long-term drift in inertial navigation systems. Overall, the bias behavior underscores the importance of initial calibration procedures to ensure sensor output accuracy across a wide range of operational modes.

### 4.5. Scale Factor

**Figure 14:**
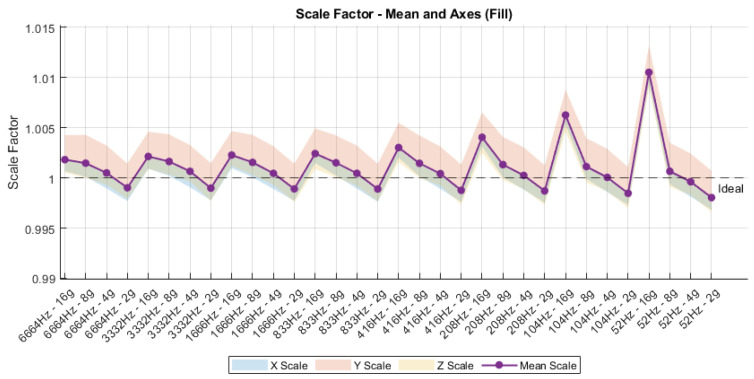
Scale Factor Sensor.

The scale factor analysis, depicted in Figure 14, provides an evaluation of the sensor’s linearity and calibration consistency across varying configurations. The ideal scale factor is defined as unity; deviations from this reference indicate either gain mismatches or calibration imperfections. Across all sampling frequencies and sensitivity ranges, the mean scale factor remains closely centered around the ideal value, with variations constrained within a narrow margin of ±0.5%. However, specific configurations—such as 1041 Hz at ±2 g and 521 Hz at ±16 g—exhibit noticeable positive deviations, potentially attributable to quantization effects or signal conditioning artifacts at those operation points. Additionally, the spread between individual axis scale factors (X, Y, and Z) suggests minimal anisotropy, further supporting the structural symmetry and stable mechanical alignment of the sensing element. These findings validate the robustness of the scale calibration over the tested configuration space, reinforcing the reliability of the IMU for precision measurement tasks.

### 4.6. Power Spectral Density

**Figure 15:**
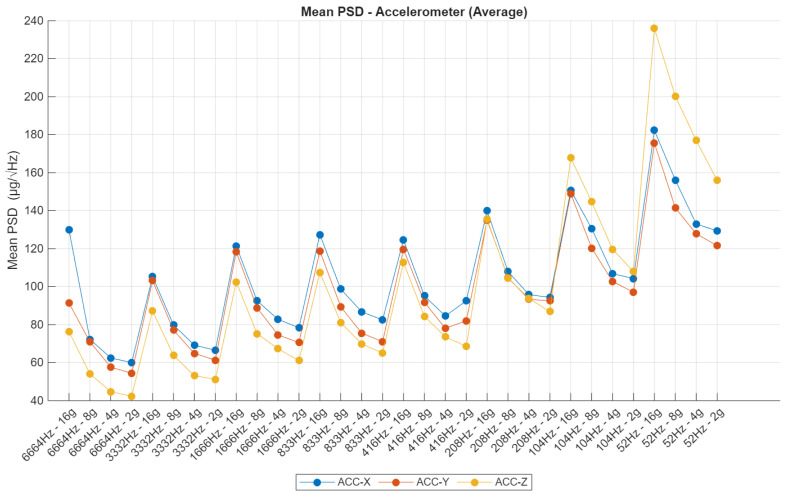
Power Spectral Density—accelerometer.

The Power Spectral Density (PSD) analysis provides a comprehensive frequency-domain assessment of the noise behavior for both the accelerometer and gyroscope across varying sampling frequencies and sensitivity configurations. For the accelerometer (Figure 15), average PSD values generally remain within a controlled range; however, a moderate increase is evident at higher sensitivity settings and reduced sampling rates. This trend is particularly pronounced at 52 Hz with ±16 g, where the Z-axis reaches peak PSD values exceeding 220 µg/sqrt(Hz), potentially due to limitations in the analog front-end or insufficient digital filtering. Across mid-range configurations—such as 416 Hz and 1666 Hz—the PSD stabilizes, and the response across all axes becomes more uniform, indicating effective signal conditioning and consistent mechanical behavior. Nonetheless, anomalies on specific axes, particularly the Z-axis at high sensitivities, highlight the need for axis-specific correction in applications requiring high accuracy.

**Figure 16:**
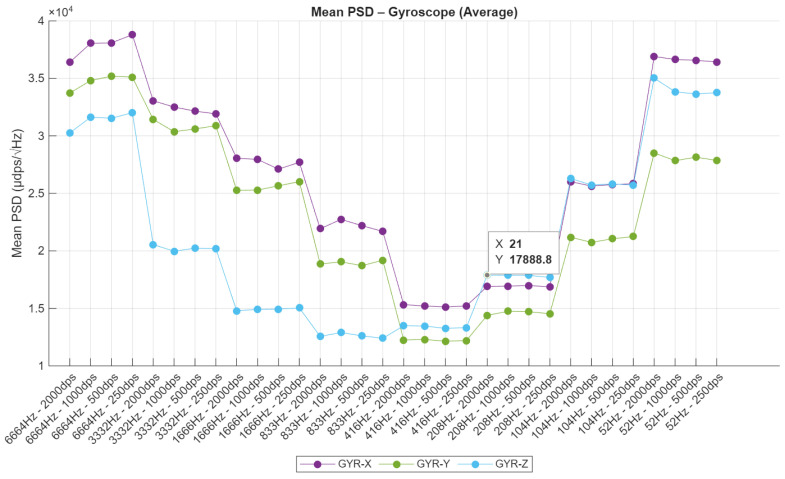
Power Spectral Density—gyroscope.

In the case of the gyroscope (Figure 16), PSD results demonstrate a more pronounced dependence on configuration, with significantly higher spectral noise observed at the highest sampling frequencies. At 6664 Hz with ±2000 dps, the X-axis reaches peak values surpassing $3.8\times 10^{4}$ µdps/sqrt(Hz), underscoring the impact of expanded bandwidth and diminished internal filtering. As the sampling frequency decreases, PSD levels drop progressively, reaching optimal performance between 208 Hz and 833 Hz for the ±1000 dps setting, where values stabilize around $1.2\times 10^{4}$ µdps/sqrt(Hz). Interestingly, a secondary rise in PSD is observed at the lowest frequencies (104 Hz and 52 Hz), which may be attributed to aliasing or limited decimation filtering. Consistently, the Z-axis exhibits the lowest noise floor, while the X-axis remains the most susceptible to noise—corroborating findings from the time-domain standard deviation analysis. Together, these results emphasize the necessity of careful configuration tuning to balance noise performance and dynamic range in inertial sensing systems.

### 4.7. Allan Deviation

The stochastic noise behavior of the inertial sensor is evaluated through Allan deviation analysis for both the accelerometer and gyroscope.

**Figure 17:**
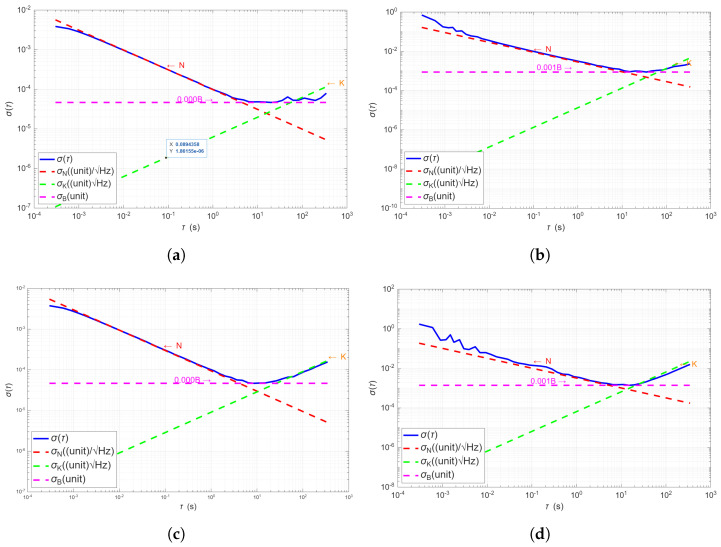

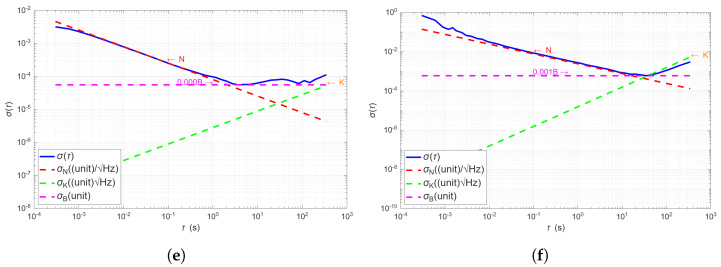
Allan deviation analysis across all sensor axes for both accelerometer (**left**) and gyroscope (**right**), with overlaid noise models. (**a**) accelerometer X axis, (**b**) Gyroscope X axis, (**c**) accelerometer Y axis, (**d**) gyroscope Y axis, (**e**) accelerometer Z axis, (**f**) gyroscope Z axis.

As shown in Figure 17a–e, the accelerometer exhibits a clear separation of noise regimes:

At short averaging times (τ<0.1s), the curves follow a slope of $-\frac{1}{2}$, indicating white Gaussian noise or Velocity Random Walk (VRW). This region is well-approximated by the red dashed lines representing $\sigma_{N}\left(\tau \right)=N/\sqrt{\tau }$. The estimated VRW noise level is on the order of $10^{-3}\;g/\sqrt{\mathrm{Hz}}$, in line with typical MEMS performance.

As τ increases, the Allan deviation plateaus due to bias instability (BI), denoted by the magenta lines. The flat shape of the deviation implies the presence of flicker noise or temperature-sensitive drift, and the BI level is approximately $10^{-4}\;g$.

At longer intervals (τ>1s), the Allan deviation increases again with a slope of $+\frac{1}{2}$, indicative of Acceleration Random Walk (ARW), shown by the green dashed lines. The ARW is especially notable in the Y-axis (Figure 17c), and is attributed to long-term drift sources such as thermal variations or mechanical stress.

Figure 17b–f present the Allan deviation for the gyroscope. At short integration times, all axes follow a $-\frac{1}{2}$ slope, corresponding to Angle Random Walk (ARW), with the red dashed lines accurately modeling this behavior. The estimated white noise values *N* fall within the expected range for MEMS gyroscopes ($10^{-3}$ to $10^{-2}{}^{\circ}/\sqrt{s}$).

The middle region, where the curve flattens, reflects bias instability. With plateaus around $10^{-3}{}^{\circ}/s$, this regime is captured by the magenta dashed lines. The origin of this component is typically associated with 1/f noise or thermal dependencies.

For τ>3s, the slope increases to $+\frac{1}{2}$, identifying the presence of Rate Random Walk (RRW). This regime is modeled by the green dashed lines ($\sigma_{K}\left(\tau \right)=K\sqrt{\tau }$). Although this behavior is especially prominent in the Z-axis (Figure 17f), it is also observable in the X and Y axes, albeit with slight deviations due to fitting limitations. The onset of RRW varies slightly across axes, suggesting the need for axis-specific fitting strategies.

In both accelerometer and gyroscope results, the experimental Allan deviation curves exhibit excellent agreement with the canonical noise models. The presence and correct identification of white noise, bias instability, and random walk in all axes confirm the sensor’s consistency and suitability for stochastic modeling. These parameters are crucial for implementing error-state propagation and optimizing navigation performance in filtering frameworks such as extended Kalman filters or inertial odometry.

## 5. Discussion

The experiments indicate that the sampling frequency is primarily limited by two factors: the SPI interface and the microcontroller’s processing speed. Although the sensor demonstrates superior sampling capabilities compared to commercial alternatives, future designs could further optimize performance by improving these aspects.

The power consumption analysis revealed that peak loads occur during data logging operations, particularly when opening and saving files to memory. Therefore, energy efficiency is closely linked to the acquisition buffer size: larger buffers reduce the frequency of write operations and thus lower the power overhead. Fine-tuning buffer size can consequently minimize memory access events and improve overall energy performance.

The PSD results exhibit discrepancies that may stem from calibration inconsistencies during production, environmental variation, or potential measurement errors. Understanding these influences is essential for a comprehensive evaluation of the sensor’s true performance. In particular, the Z-axis consistently exhibited higher PSD values and larger bias fluctuations. This behavior may arise from mounting asymmetry in the test fixture, axis-specific imperfections in the analog front-end circuitry, or residual calibration errors. To mitigate these effects, future implementations could include axis-specific calibration routines, as well as digital filtering approaches optimized for the Z-axis. Mechanical refinements in the mounting and packaging are also expected to reduce asymmetry and improve cross-axis consistency.

Based on the findings, three design refinements are recommended. First, the sensor-to-controller interface should adopt a dedicated SPI line, while the storage module should employ the SDIO interface to avoid data throughput bottlenecks. Second, replacing the BMS with an LDO and delegating battery management to the microcontroller would reduce power consumption and thermal interference. Third, the removal of the SD card socket simplifies the design, reduces cost, and improves enclosure hermeticity.

Table 2 compares Bimu R2 with other commercial IMU devices, including NilsPod [15], Shimmer 3 [16], Physilog^®^5 [17], and XSens Dot [18]. Bimu stands out for its compact size (45.5 × 24 × 9.7 mm), 32 GB onboard storage—the largest among the devices—and a sampling rate of up to 4000 Hz. It also offers the second-highest battery capacity (220 mA·h), supporting extended recordings. Although sensor fusion is not integrated, future firmware updates may incorporate advanced features such as gait or balance analysis.

Unlike existing commercial IMUs, Bimu R2 incorporates specific refinements to overcome common limitations. For instance, compared to the NilsPod system [15], which is optimized for long-term monitoring but exhibits synchronization constraints in multi-sensor setups, Bimu R2 implements a dedicated synchronization interface ensuring sub-millisecond alignment across modules. In practice, Bimu R2 allows synchronization of up to four units via the Bimu-Dock base station, or alternatively through a direct wired connection, guaranteeing precise timing even in distributed sensor networks. In contrast to Shimmer 3 [16], which is widely used in biomechanics but prone to thermal drift in extended sessions, Bimu R2 adopts a low-noise LDO-based design to reduce temperature-induced bias instability. Similarly, while the Physilog^®^5 platform [17] provides robust clinical-grade kinematic tracking, its resilience in high-frequency scenarios (>200 Hz) is limited; Bimu R2 is specifically optimized for stable acquisition in such high-dynamic conditions. Finally, compared to XSens Dot [18], which prioritizes wearability and ease of use at the cost of raw data throughput, Bimu R2 leverages a dedicated SPI/SDIO architecture to ensure continuous high-bandwidth streaming without data loss. These distinctions highlight Bimu R2 as a versatile platform addressing synchronization, thermal stability, and high-frequency robustness beyond current commercial solutions.

This study presents the design and characterization of a wearable IMU with potential applications in healthcare and sports monitoring. The device demonstrates strong performance in terms of energy efficiency and data storage; however, further optimization is required in sensor connectivity and battery management.

Noise analysis revealed low variability across most axes, although the Z-axis showed inconsistencies that warrant further investigation. Bias measurements confirmed minimal error in the accelerometer, while the gyroscope presented more notable deviations. Scale factor testing validated the sensor’s accuracy, with results closely aligning with expected values.

These findings underscore the importance of tailoring sensor specifications to specific use cases. The detailed evaluation of noise, bias, and scaling factors presented here provides a valuable framework for researchers developing or refining wearable IMU systems. As wearable technologies continue to evolve, future work should aim to extend these improvements to broader application domains.

## 6. Conclusions

This work presented the third-generation design and characterization of Bimu, a wearable IMU specifically developed for high-frequency motion capture. The new version (Bimu R2) integrates several key improvements: (i) a simplified power architecture with an LDO regulator, reducing power consumption and thermal interference; (ii) a rigid PCB design with steel reinforcement, enhancing thermal stability and precision; (iii) removal of the external SD socket in favor of onboard memory, improving mechanical robustness and hermeticity; and (iv) dedicated synchronization interfaces, enabling sub-millisecond alignment of up to four modules via Bimu-Dock or direct wired connection. Experimental results demonstrated reduced drift, low noise variability, and stable performance at sampling rates above 200 Hz, validating the device for demanding biomechanical applications.

Beyond these achievements, Bimu R2 establishes a platform that can be extended in several research directions. Future work will include systematic validation in clinical and rehabilitation contexts, the development of firmware-level sensor fusion algorithms, and large-scale synchronized deployments for sports performance analysis and impact biomechanics. These pathways will further consolidate Bimu as a versatile tool bridging laboratory-grade measurement with field-ready usability.

As shown in Table 1, the STM32U031K4 used in this work offers a significantly higher core frequency (56 MHz vs. 32 MHz), lower active current per MHz (≈36 µA/MHz vs. ≈87 µA/MHz), and a faster ADC (2.5 MSPS vs. 1 MSPS) compared to the STM32L051K6 used in previous versions. These improvements translate into greater processing headroom, better energy efficiency at high sampling rates, and more responsive data acquisition—all of which are particularly advantageous for high-frequency motion capture applications.

## Figures and Tables

**Table 1:** Comparison of MCUs used in Bimu prototypes. The STM32U031K4 is used in the present study, while the STM32L051K6 was used in previous versions.

Feature	STM32U031K4 (This Work)	STM32L051K6 (Previous Work)
Core	ARM Cortex-M0+ @ 56 MHz	ARM Cortex-M0+ @ 32 MHz
Flash Memory	32 KB	64 KB
SRAM	8 KB	8 KB
Operating Voltage	1.71–3.6 V	1.8–3.6 V
Active Current (Run, per MHz)	≈36 μA/MHz	≈87 μA/MHz
Low-Power Modes	Stop 2, Standby	Stop, Standby
GPIO Count	up to 37	up to 55
Timers	6 (16-bit)	7 (16/32-bit)
ADC	12-bit, 2.5 MSPS	12-bit, 1 MSPS
Communication Interfaces	I2C, SPI, USART, LPUART	I2C, SPI, USART, LPUART
Packages	LQFP32, UFQFPN32	LQFP32, UFQFPN32

**Table 2:** Comparison of Bimu R2 with existing commercial IMUs.

System	Synchronization	Thermal drift stability	High-frequency resilience
NilsPod [15]	Limited multi-sensor synchronization	Moderate	Suitable up to medium frequencies (100–200 Hz)
Shimmer 3 [16]	External triggers available but prone to offsets	Affected in extended sessions	Reliable below 200 Hz
Physilog^®^5 [17]	Clinical-grade synchronization	Stable	Limited above 200 Hz
XSens Dot [18]	Easy Bluetooth sync, not precise for research	Good, optimized for comfort	Limited raw throughput in high dynamics
**Bimu R2 (this work)**	Hardware trigger via Bimu-Dock (up to 4 units) or wired bipolar cable; sub-ms precision	LDO design reduces bias instability	Optimized for stable capture above 200 Hz

## Data Availability

The data presented in this study are available on request from the corresponding author.

## Abbreviations

The following abbreviations are used in this manuscript:

ADC	Analog-to-Digital Converter
ARW	Angular Random Walk
BMS	Battery Management System
FIFO	First In, First Out
IMU	Inertial Measurement Unit
LDO	Low Dropout Regulator
MDPI	Multidisciplinary Digital Publishing Institute
NTC	Negative Temperature Coefficient
PCB	Printed Circuit Board
PSD	Power Spectral Density
RAM	Random Access Memory
RRW	Rate Random Walk

## References

[1] Alvarez J.C., Álvarez D., López A.M. (2018). Accelerometry-based distance estimation for ambulatory human motion analysis. Sensors.

[2] Studenski S., Perera S., Patel K., Rosano C., Faulkner K., Inzitari M., Brach J., Chandler J., Cawthon P., Connor E.B. (2011). Gait speed and survival in older adults. JAMA.

[3] Afilalo J., Eisenberg M.J., Morin J.-F., Bergman H., Monette J., Noiseux N., Perrault L.P., Alexander K.P., Langlois Y., Dendukuri N. (2010). Gait speed as an incremental predictor of mortality and major morbidity in elderly patients undergoing cardiac surgery. J. Am. Coll. Cardiol..

[4] Tao W., Liu T., Zheng R., Feng H. (2012). Gait analysis using wearable sensors. Sensors.

[5] Patrizi G., Ciani L., Catelani M., Sommella P., Pietrosanto A. (2024). Temperature Sensitivity Analysis of Inertial Measurement Unit Under Dynamic Conditions. IEEE Trans. Instrum. Meas..

[6] Gu H., Zhao B., Zhou H., Liu X., Su W. (2019). MEMS Gyroscope Bias Drift Self-Calibration Based on Noise-Suppressed Mode Reversal. Micromachines.

[7] Rosano C., Rosano C., Boudreau R.M., Simonsick E.M., Ferrucci L., Sutton-Tyrrell K., Hardy S.E., Atkinson H.H., Yaffe K., Satterfield S. (2008). Executive function, memory, and gait speed decline in well-functioning older adults. J. Am. Geriatr. Soc..

[8] Hausdorff J.M. (2001). Gait dynamics, fractals and falls: Finding meaning in the stride-to-stride fluctuations of human walking. J. Appl. Physiol..

[9] Brach J.S., Perera S., Studenski S., Katz M., Hall C., Verghese J. (2010). Meaningful change in measures of gait variability in older adults. Gait Posture.

[10] Hayes T.L., Hagler S., Austin D., Kaye J., Pavel M. Unobtrusive assessment of walking speed in the home using inexpensive PIR sensors. Proceedings of the Annual International Conference of the IEEE Engineering in Medicine and Biology Society (EMBS).

[11] Caby B., Kieffer S., de Saint Hubert M., Cremer G., Macq B. (2011). Feature extraction and selection for objective gait analysis and fall risk assessment by accelerometry. Biomed. Eng. Online.

[12] Mitschke C., Zaumseil F., Milani T.L. (2017). The Influence of Inertial Sensor Sampling Frequency on the Accuracy of Measurement Parameters in Rearfoot Running. Comput. Methods Biomech. Biomed. Eng..

[13] Aubol K.G., Milner C.E. (2023). Minimum Sampling Frequency for Accurate and Reliable Tibial Acceleration Measurements During Rearfoot-Strike Running in the Field. J. Appl. Biomech..

[14] Valdés-Tirado D., García G., Álvarez J. (2024). Design and Characterization of a Wearable Inertial Measurement Unit. Sensors.

[15] Zhou H., Hansen C., Lund M. (2020). NilsPod: An Open-Source Wearable IMU Platform for Ergonomic and Biomechanical Studies. Sensors.

[16] Burns A., Greene B.R., McGrath M.J. SHIMMER™: A Wireless Sensor Platform for Non-Invasive Biomedical Research. Proceedings of the IEEE EMBC.

[17] Dadashi F., Mariani B., Rouhani H., Aminian K. (2018). Physilog 5: Clinical-Grade Wearable Inertial Sensor for Motion Analysis. Front. Bioeng. Biotechnol..

[18] Xsens Technologies (2020). Xsens DOT: Wearable Sensor Platform for Human Motion Measurement. https://www.movella.com/hubfs/Downloads/Whitepapers/Xsens%20DOT%20WhitePaper.pdf.

[19] Valdés-Tirado D., García G., Álvarez J., López A., Álvarez D. Design of an IMU Based Wearable Measurement System for Rapid Events. Proceedings of the IEEE International Instrumentation and Measurement Technology Conference (I2MTC).

